# Rhabdomyosarcoma: Advances in Molecular and Cellular Biology

**DOI:** 10.1155/2015/232010

**Published:** 2015-09-01

**Authors:** Xin Sun, Wei Guo, Jacson K. Shen, Henry J. Mankin, Francis J. Hornicek, Zhenfeng Duan

**Affiliations:** ^1^Department of Orthopaedic Surgery, Massachusetts General Hospital, 100 Blossom Street, Boston, MA 02114, USA; ^2^Sarcoma Biology Laboratory, Center for Sarcoma and Connective Tissue Oncology, Massachusetts General Hospital, 100 Blossom Street, Boston, MA 02114, USA; ^3^Department of Orthopaedic Oncology, Peking University People's Hospital, 11 Xizhimen South Street, Xicheng District, Beijing 100044, China

## Abstract

Rhabdomyosarcoma (RMS) is the most common soft tissue malignancy in childhood and adolescence. The two major histological subtypes of RMS are alveolar RMS, driven by the fusion protein PAX3-FKHR or PAX7-FKHR, and embryonic RMS, which is usually genetically heterogeneous. The prognosis of RMS has improved in the past several decades due to multidisciplinary care. However, in recent years, the treatment of patients with metastatic or refractory RMS has reached a plateau. Thus, to improve the survival rate of RMS patients and their overall well-being, further understanding of the molecular and cellular biology of RMS and identification of novel therapeutic targets are imperative. In this review, we describe the most recent discoveries in the molecular and cellular biology of RMS, including alterations in oncogenic pathways, miRNA (miR), *in vivo* models, stem cells, and important signal transduction cascades implicated in the development and progression of RMS. Furthermore, we discuss novel potential targeted therapies that may improve the current treatment of RMS.

## 1. Introduction

Rhabdomyosarcoma (RMS) is the most prevalent soft tissue tumor in children and adolescents, accounting for 5% of all pediatric tumors [1, 2]. It is estimated that 350 new cases of RMS are diagnosed each year in patients under 20 years of age in the United States [2]. In contrast, RMS is extremely rare in adults. There is a slight male predominance (1.4 times more common in males than in females), but there are no significant differences in the incidence rates among races or different ethnic groups [3]. As RMS is derived from primitive mesenchymal stem cells directed towards myogenesis, it can arise in a variety of anatomic sites throughout the body [4]. RMS can occur either as a primary malignancy or as a component of a heterogeneous malignancy, such as a malignant teratomatous tumor [5]. Additionally, a small percentage of cases are associated with known genetic disorders, such as neurofibromatosis type 1 and the Li-Fraumeni familial cancer syndrome [6].

The World Health Organization (WHO) recently revised the classification of RMS subtypes as alveolar rhabdomyosarcoma (ARMS), embryonal rhabdomyosarcoma (ERMS), pleomorphic rhabdomyosarcoma (PRMS), and sclerosing/spindle cell rhabdomyosarcoma (SRMS) in 2013 [7]. ARMS is a high-grade malignancy occurring mostly in adolescents and young adults. The most common site for ARMS is in the deep tissue of extremities. ERMS represents approximately 70% of all childhood RMS, usually afflicting infants or children under 10 years of age. ERMS often affects the head and neck regions, especially the orbit. PRMS usually occurs in adult males in the deep tissue of extremities but may occur at any site. In adult patients, the pleomorphic variant is associated with the worst prognosis [8].

Since the 1970s, the Intergroup Rhabdomyosarcoma Study Group (IRSG) has conducted a series of clinical trials comparing risk-base and has established a series of treatment guidelines [9]. Currently, multidisciplinary management including chemotherapy and surgery with or without radiation has become the standard treatment for RMS. The 5-year survival rate of RMS has increased from 25% in 1970 up to 60% since 2000 [1, 10]. However, there has been little improvement in the oncological outcome of patients with RMS in recent years. Drug resistance and metastatic disease represent the two most common phenomena for therapy failure. Some randomized chemotherapy trials have failed to improve outcome despite the introduction of newer or more intensive therapies. Thus, there is an urgent need for alternative, more effective treatment strategies. Recent molecular and genetic analysis of these tumors has produced substantial new insights into molecular cell biology, molecular cytogenetics, and tumorigenesis of RMS, leading to a better understanding of RMS development at the molecular level. These advances may ultimately lead to better clinical understanding and to potentially developing more potent targeted therapies. The purpose of this review is to summarize these most recent findings in RMS.

## 2. Novel Discoveries of Chromosomal Alterations in RMS

Malignant transformation occurs cytogenetically due to the accumulation of somatic mutations by the acquisition of tumor-specific chromosomal translocations.

### 2.1. Gains and Losses of Chromosomes in RMS

Comparative Genomic Hybridization (CGH) analysis has revealed that all RMS have specific gains and losses [11–18] (Table 1). ERMS frequently exhibits gains or losses of specific whole chromosomes, whereas ARMS is characterized by the presence of regions of genomic amplification [11, 19]. The focal regions and genes, most of which have frequent gains and amplifications, include 12q13.3–q14.1 and 8p11.2–11.2 and CDK4, MYCN, GLI, MDM2, FGFR1, and FGFR4, respectively. Frequently, the genes differentially expressed in subtypes of RMS, particularly when they are from chromosomal regions, show a high level of gains in cell lines. Previous CGH studies have shown that ARMS tumors tend to have fewer copy number variants than ERMS tumors [12, 20]. For example, frequent gains were detected in TYROBP, HCST, LRFN3, and ALKBH6 (19q13.12) in ERMS, but not in ARMS [15]. These studies have identified a number of genetic alterations in RMS. Many of these chromosomal changes may be responsible for tumor progression and proliferation. These genetic alterations may be potential treatment targets in RMS [11].

### 2.2. Chromosomal Translocations in ARMS

Chromosomal analyses have demonstrated two translocations associated with ARMS, t(2;13)(q35;q14) and t(1;13)(p36;q14) [21]. Initial studies detected these two gene fusions in 80% of ARMS [22]. These characteristic chromosomal translocations are adjacent to the 5′ DNA-binding domains of PAX, a member of the paired box transcription factor family, and the transactivation domain at the 3′ end of FKHR, a member of the forkhead/HNF-3 transcription factor family. Approximately 75% of these structural rearrangements translocate the PAX3 gene at 2q35 to the FKHR gene at 13q14, as t(2;13)(q35;q14); less frequently, in the other 25%, the t(1;3)(q36;q14) translocation fuses PAX7 to FKHR [23]. Previous studies have demonstrated that PAX3/FKHR fusion gene status significantly improves current risk stratification, while the presence of PAX7/FKHR and rarer variant fusion gene products require further investigation [24]. The remaining 20% of ARMS is* PAX* gene fusion-negative (PFN) and forms a more heterogeneous group, which remains a challenge to detect due to the lack of consistent chromosomal rearrangements. PFN ARMS has a similar clinical course to ERMS, which suggests that the fusion gene status provides more accurate information about patient outcomes than did the histologic subtype. Despite the low overall burden of somatic mutations in fusion-positive RMS, multiple genes were recurrently altered, including NRAS, KRAS, HRAS, FGFR4, PIK3CA, CTNNB1, FBXW7, and BCOR [25].

**Figure 1:**
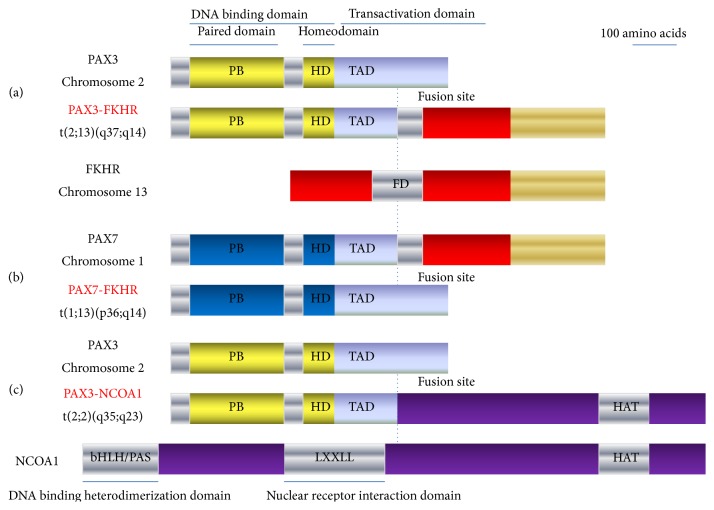
The chromosomal rearrangements in ARMS. 80% of ARMS classified as translocation-positive ARMS carry characteristic chromosomal translocations demonstrated as t(2;13)(q35;q14), t(1;13)(p36;q14), and (2;2)(q35;p23). In (a) and (b), the translocations fuse the FKHR (a member of the forkhead/HNF-3 transcription factor family) locus on chromosome 13 to either PAX3 on chromosome 2 or PAX7 on chromosome 1. In (c), the translocation generated a fusion protein composed of PAX3 and the nuclear receptor coactivator NCOA1, having similar transactivation properties as PAX3/FKHR.

Another novel translocation, t(2;2)(q35;p23), was identified in ARMS biopsy samples by gene expression signatures [26]. The chromosomal translocation generates a fusion protein composed of PAX3 and the nuclear receptor coactivator NCOA1, which has similar transactivation properties as PAX3/FKHR [26]. These biologic effects contribute to tumorigenesis by modulating myogenic differentiation, altering growth and apoptotic pathways, and stimulating motility and other metastatic pathways (Figure 1).

### 2.3. Chromosomal Alterations in ERMS

ERMS exhibits a loss of imprinting (LOI), leading to a twofold gene dosage effect [27]. Most tumors have at least one 15-Mb region with loss of heterozygosity (LOH) along chromosome 11 [27]. The allelotype of ERMS demonstrates a high frequency of LOH on chromosomes 11p, 11q, and 16q [28]. ERMS tumorigenesis can result from the inactivation of the parental bias of chromosome 11p15, which is the most common rearrangement in ERMS [29]. The proportion of ERMS with LOH along chromosome 11 is considerably higher than in other subtypes [27]. In histopathological analysis, ERMS expresses low PAX3 levels and elevated PAX7 levels [30]. Hosoi et al. identified a hidden 2q35 breakpoint as a novel PAX3 rearrangement in complex chromosomal translocations in ERMS [31].

### 2.4. Chromosomal Alterations in Other RMS

There are limited studies on the biological pathways involved in other subtypes of RMS, compared with the two major subtypes ARMS and ERMS. Fluorescence In Situ Hybridization (FISH) reveals amplification of JUN (1p31), MYC (8q24), CCND1 (11q13), INT2 (11q13.3), MDM2 (12q14.3–q15), and MALT (18q21) in these tumor cells, contributing to the pathogenesis of PRMS [32]. MYOD1 homozygous mutations are also reported as frequent, recurrent, and pathognomonic events in adult-type SRMS [33, 34] (Table 2). Furthermore, four out of five pediatric tumors showed MYOD1 mutations in a study [34]. Yoshida et al. described a 8q13 locus (NCOA2) gene rearrangement in a small subset of SRMS occurring uniquely in the infantile/congenital setting, fused with key transcription factors involved in skeletal muscle differentiation, such as SRF and TEAD1 [35]. They also identified NCOA2 as a candidate PAX3 partner gene. The PAX3-NCOA2 fusion gene plays a dual role in the tumorigenesis of RMS, promotion of the proliferation, and inhibition of the myogenic differentiation of RMS cells [35]. As PAX3-NCOA2-induced tumors grow more slowly, SRMS that are PAX3-NCOA2 fusion-positive could be associated with a very favorable prognosis [36].

Not all RMSs occur as sporadic primary tumors. Occasionally, RMS inherits a mutant gene as part of an established familial syndrome. For example, Beckwith-Wiedemann Syndrome (BWS) with RMS involves dysregulation or alteration of imprinted genes in the 11p15.5 chromosomal region, including IGF2, H19, and CDKN1C (p57/KIP2) [37].

## 3. Cell of Origin in RMS

There have been a number of studies aimed at deciphering the cell origin for RMS [56–61].

### 3.1. Myogenic Differentiation in the Tumorigenesis

**Figure 2:**
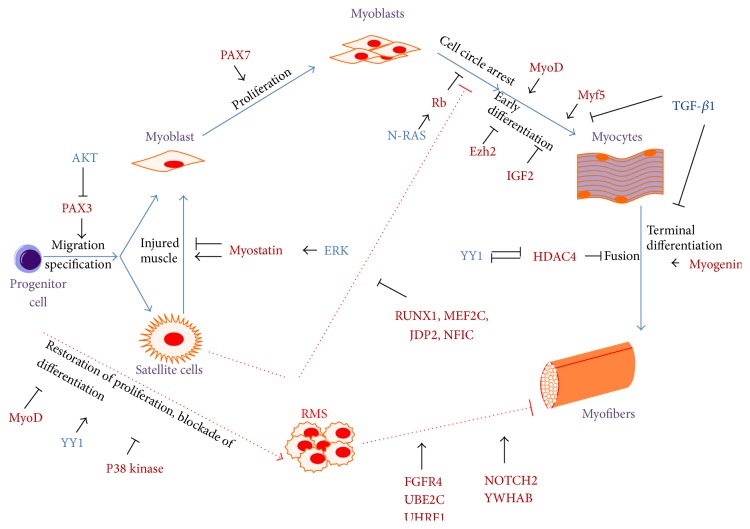
Myogenic pathways in the tumorigenesis of RMS. In aberrant neoplastic condition, progenitor cells residing in muscle result in aberrant pathways, which lead to malignant transformation and fail to differentiate, proliferate uncontrollably, and form RMS. Sharp arrows (→) indicate upregulation/activation and blunt arrows (⊥) indicate downregulation/inhibition.

Several studies have focused on elucidating the mechanisms governing the impaired myogenic program in RMS [62, 63] (Figure 2). RMS originates as a consequence of regulatory disruption of the growth and differentiation of myogenic precursor cells. Progenitor cells reside in muscle and their activation results in either proper myogenesis or aberrant signaling pathways leading to the development of RMS. Based on the skeletal muscle lineage, a complete transcriptome analysis of RMS was performed to compare normal and fetal muscles [56]. The high degree of similarity between fetal muscle and RMS expression profiles reflects the undifferentiated myogenic nature of RMS. The genes exclusively upregulated in RMS, including FGFR4, NOTCH2, UBE2C, UHRF1, and YWHAB genes, contribute to the failure of RMS cells to complete normal skeletal muscle development and progress to an alternative fate [56]. RMS cells represent an arrested state in the development of normal skeletal muscle, with regional silencing of differentiation factors leading to the maturation defect in RMS [57]. The expression pattern of muscle-specific proteins regulating myogenic differentiation has been extensively examined in RMS. The family of myogenic transcription factors (MYOD, MYF5, myogenin, and MRF4) are considered to be responsible for the determination of stem cells into myoblasts and differentiation into myocytes [58, 59]. All these factors are activated during the onset or progression of myogenesis and are subsequently silenced to reach a final muscular differentiation. There are statistical differences in the different subtypes of RMS tumors with MYOD or myogenin staining patterns. Amplification of MDM2 in an RMS cell line interferes with MYOD activity and consequently inhibits overt muscle cell differentiation [61]. Interleukin-4 receptor (IL-4R) has also been proposed to be important for the maturation of myotubes [64]. An IL-4R blockade might help therapeutically to modulate the expression of myogenic transcription factors (MYOD or myogenin) [57, 64].

### 3.2. Potential Cancer Stem Cell in RMS

Cancer Stem Cells (CSCs) may contribute to inherent refractory responses to current therapies and metastasis [65]. Many CSC models play an important role in the development of RMS [38–41, 66] (Table 3).

A study explored a potential important role for mesenchymal stem cells (MSCs) as the cell of origin of ARMS [67]. Ren et al. confirmed that PAX-FKHR fusion genes commit MSCs to a myogenic lineage by inhibiting terminal differentiation and contributing to ARMS formation [39]. Another study showed that PAX-FKHR induces skeletal myogenesis in MSCs by transactivating MYOD and myogenin and transforms mesenchymal progenitor cells to the skeletal muscle lineage leading to malignant formation resembling ARMS [39].

As a human hematopoietic CSC marker, Walter et al. proposed that upregulated CD133 in ERMS can act as a prognostic marker and might help with the development of novel targeted therapies for ERMS [38].

## 4. Signaling Pathway Alterations in RMS

**Figure 3:**
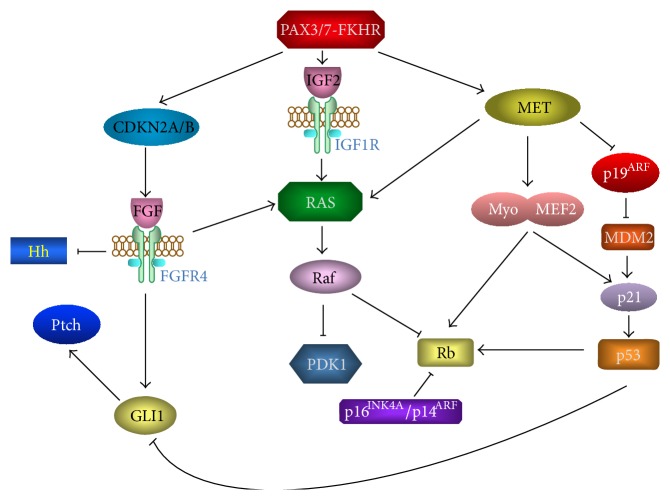
Genetic analyses of RMS have pinpointed several common alterations, including inactivation of a master regulator of p53 and Rb pathways, CDKN2A/B, and activation of FGFR4, RAS, and Hedgehog (Hh) signaling. The modifications of these pathways influence oncogenesis and metastatic potential.

Recent studies have looked into signaling pathway alterations and their downstream effects in RMS. These new findings not only helped to improve the understanding of this malignancy but also offered novel potential therapeutic strategies for improved treatment for patients with RMS [68, 69] (Figure 3).

### 4.1. RAS Signaling Pathway

RAS mutations commonly maintain the protein in its GTP bound state and therefore render it constitutively active in RMS. Zhang et al. identified RAS family members NRAS, KRAS, and HRAS in the RAS pathway as the most commonly mutated genes in ERMS [70]; the mutation status of these genes is significantly associated with the risk of ERMS development [70]. A study found that a majority of ERMS demonstrated activation of the RAS pathway exclusively either by homozygous deletion of NF1 or by point mutations in one of the RAS family members as described above [13]. Despite remarkable genetic and molecular heterogeneity, most (93%, 41/44) RMS tumors hijacked a common receptor tyrosine kinase/RAS/PIK3CA genetic axis [25]. It could occur via two alternative mechanisms: rearrangement of a PAX gene and accumulation of mutations that were downstream targets of the PAX fusion protein.

Skeletal muscle cells have a robust antioxidant defense system to protect the DNA, lipids, and proteins from the deleterious effects of excess Reactive Oxygen Species (ROS). Cancer cells also have elevated ROS due to their increased metabolic activity, oncogenic stimulation, and mitochondrial dysfunction. These findings implicate RAS mutations and oxidative stress as potential therapeutic targets for high-risk ERMS.

### 4.2. IGF Signaling Pathway

There is clear preclinical data that supports the involvement of the Insulin-like Growth Factor (IGF) signaling pathway in RMS tumorigenesis and progression [69]. Zhu and Davie demonstrated that IGF promotes the proliferation of RMS cells, while blocking IGF signaling interferes with cell growth* in vivo*. The IGF receptor, IGF-1R, is highly overexpressed both on the cell surface and in the nucleus of RMS cells; furthermore, RMS cell lines are sensitive to IGF-1R inhibition. The IGF-2 locus shows a loss of imprinting in both ERMS and ARMS tumors, and the expression of PAX3-FKHR has been demonstrated to upregulate IGF-2 and activate IGF pathway in ARMS [69]. Marianna et al. found that IGF-2 and tumor suppressors p19Arf and p21Cip1 are overexpressed and prominently upregulated in RMS mouse models [71].

### 4.3. TGF-*β* Signaling Pathway

The regulatory role of Transforming Growth Factor-*β* (TGF-*β*) in RMS has been recently studied [72]. Wang et al. demonstrated that the expression of TGF-*β*1 is significantly higher in RMS than in normal skeletal muscle. The inhibition of TGF-*β*1 expression by shRNA-expressing vectors reverses the malignant behavior of RMS by inhibiting cell growth and inducing myogenic differentiation. TGF-*β*1 shRNA induces myogenin expression, a regulator of myogenic differentiation genes. Myogenin acts as a target for negative regulation of myogenesis by TGF-*β*1 signals with a differentiation regulatory cascade. These results suggest that the TGF-*β*1 signaling pathway disrupts the differentiation of myogenic progenitors, leading to the development of RMS.

### 4.4. FGF Signaling Pathway

Fibroblast Growth Factor (FGF) is crucial in embryonic development and functions to drive proliferation. FGFs and their receptors (FGFRs) are essential regulators in the processes of proliferation, antiapoptosis, drug resistance, and angiogenesis in RMS [69]. RMS overexpresses the receptor tyrosine kinase FGFR4, which causes autophosphorylation and constitutive signaling in correlation with poor differentiation and decreased survival [73]. Recent work has shown that FGF signaling can prevent ARMS cells from apoptosis induced by targeting the IGF1-R-PI3K-mTOR pathway [74].

### 4.5. ERK Signaling Pathway

Previous studies have shown that elevated myostatin expression deregulates Extracellular Regulated Kinase (ERK) signaling and deficient activation of the p38 pathway contributes to the differentiation block in RMS cells [62]. The ERK pathway is frequently highly activated in RMS cells from embryonic derivation due to the presence of activating RAS mutations, leading to reduced differentiation by blocking the p38 pathway [75]. These findings indicate that interventions that target the myostatin/ERK/p38 network could be an effective way to promote differentiation of RMS.

### 4.6. Hippo Signaling Pathway

Many upstream signal transduction proteins, in response to cues from the cellular microenvironment, regulate the core Hippo pathway [76]. The Hippo pathway may limit tumorigenesis by inducing the cytosolic localization of the transcriptional cofactor Yes-Associated Protein 1 (YAP1). High YAP levels and activities increase activated satellite cells and prevent differentiation by activating genes that inhibit differentiation [77]. YAP1-TEAD1 was found to upregulate proproliferative and oncogenic genes and maintain the ERMS differentiation block by interfering with MYOD1 and MEF2 prodifferentiation activities [76]. Inhibition of the Hippo pathway is exhibited in RMS through downregulation of Hippo pathway tumor suppressors or upregulation of YAP [78]. These data suggest that Hippo pathway dysfunction promotes RMS development.

### 4.7. Notch Signaling Pathway and Wnt Signaling Pathway

Notch pathway, as an embryonic signaling pathway, promotes muscle stem cell maintenance by inhibiting myogenic early differentiation and expanding pools of progenitor cells during both embryogenesis and postnatal muscle regeneration. Similar to Notch, Wnt has various roles in embryonic, fetal, and neonatal myogenesis during skeletal myogenesis. As both pathways are regulators of myogenic lineage determination and maturation, RMS can arise from disordered regulation of these normal developmental pathways [79].

### 4.8. Caveolins in RMS

Recent findings have shown that caveolins are expressed in a cell stage-dependent manner in RMS [80, 81]. Caveolins are scaffolding proteins that regulate several targets and pathways (such as p53, IGF, RAS/ERK, and TGF-*β*/myostatin signaling) associated with RMS development, and therefore loss or gain of caveolin function is able to generate multiple effects on the tumor behavior. Therefore, recognizing their precise roles in RMS progression will be crucial to elaborate targeted therapies; particularly, as one of the three different isoforms, CAV1, could act as a potent tumor suppressor in ARMS. Inhibition of CAV1 function can contribute to aberrant cell proliferation, leading to ARMS development [80].

### 4.9. DNA Repairing System

**Figure 4:**
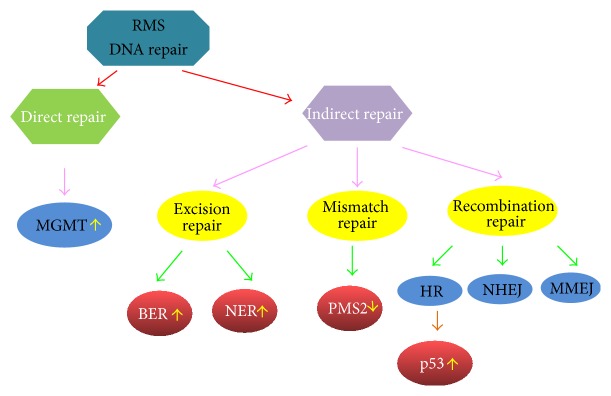
The DNA repair systems in RMS. There are two pathways repairing the DNA lesions, directly without affecting DNA structure and indirectly by DNA phosphodiester backbone cleavage. The modifications in DNA repair enzymes expression or activity lead to resistance to chemotherapy and radiation in RMS tumor cells. MGMT, O^6^-methylguanine-DNA methyltransferase; BER, base excision repair; NER, nucleotide excision repair; HR, homologous recombination; NHEJ, nonhomologous end-joining; MMEJ, microhomology-mediated end-joining.

Some studies indicate that RMS is characterized by germline mutations of DNA repair genes [82, 83]. DNA repair gene alterations in RMS occur secondarily to malignant transformation. The modifications in DNA repair enzyme activity can result in resistance to adjuvant treatment in RMS tumor cells, which limits the prognosis of RMS. There are two pathways for repairing DNA breaks: directly without affecting DNA structure and indirectly by DNA phosphodiester backbone cleavage [82] (Figure 4). Direct repair includes repair during replication and enzymatic repair. Indirect repair is comprised of excision repair systems, including base excision repair (BER), nucleotide excision repair (NER), mismatch repair (MMR), and recombination repair (RR) [83]. Defective DNA repair mechanisms result from point mutations or LOH in RMS and contribute to tumorigenesis. MGMT and MMR protein activity and expression levels are used as predictor indices for therapy outcome in RMS. BER, as the major DNA repair system against damage resulting from cellular metabolism, can reverse the cytotoxic effects of alkylation agents used, such as antineoplastics; subsequent tumor progression and death implicates an RMS mechanism of resistance to chemotherapeutic agents. This may provide novel individualized therapeutics targeting downregulation of activated DNA repair enzymes or upregulation of DNA repair deficiencies [82].

## 5. MicroRNA (miRNA, miR) Expression in RMS

Muscle-specific and ubiquitously expressed miRs appear downregulated in RMS tumors and cell lines compared with the normal counterparts. These miRs often play crucial roles as antioncogenes. Inhibition of these miRs contributes to enhanced tumorigenesis through the modulation of diverse molecular pathways. Upregulation of prooncogenic miRs has been detected in RMS recently as well [84] (Table 4).

### 5.1. Antioncogenic miR in RMS

Gain-of-function experiments have demonstrated that reexpression of selected “tumor suppressor” miRs impairs the tumorigenic behavior of RMS cells [84, 85]. As myo-miR family members, miR-1, miR-133a, and miR-206 have been demonstrated to be downregulated in RMS and shown to have activity on mRNA expression by targeting c-Met [42, 43, 86]. Inhibition of these myo-miRNAs may cause aberrant cell proliferation and migration in myogenesis, leading to RMS development, especially for ERMS. There have been more miRs found as tumor suppressors in this malignancy, including miR-29, miR-450b-5p, miR-203, and miR-214 [44, 46, 47, 84, 87]. They are all significantly downregulated in RMS compared with normal skeletal muscles and function to inhibit tumor cell growth and induce myogenic differentiation and apoptosis in RMS.

### 5.2. Oncogenic miRNA in RMS

The transcription factor EGR1 is a tumor suppressor gene that is downregulated in RMS [48]. miR-183 functions as an oncogene by targeting EGR1 and promoting tumor cell migration. Either by direct anti-miR treatment or by indirect mechanisms that decrease transcript, miR-183 targeted treatments might provide a potential option to RMS patients.

The basic strategy of current effective miR-based treatment studies is either efficient reexpression of miRs or restoring the function of miRs to inhibit the expression of certain protein-coding genes and promote muscle differentiation. Moreover, miR expression profiling in tumors and, possibly, their detection in peripheral blood during treatment may be able to predict the response to chemotherapy or radiotherapy and be useful as a prognostic signature for the development of treatment resistance. The prognostic value of miR expression levels in RMS is a powerful tool in creating a better-tailored strategy for particular subsets of patients.

### 5.3. Long Noncoding RNAs in RMS

Long noncoding RNAs (LNCRNAs) are abundant in the mammalian transcriptome and have been shown to play a role in RMS development [88, 89]. The H19 gene, localized within a chromosomal region on human chromosome 11p15, encodes an imprinted untranslated RNA [90]. H19 opposite tumor suppressor (HOTS), a tumor growth inhibitor, is encoded by an imprinted H19 antisense transcript. Overexpression of HOTS inhibits RMS tumor cell growth. Silencing HOTS by RNAi was shown to increase* in vitro* colony formation, as well as* in vivo* tumor growth of RMS [90].

## 6. *In Vivo* Models of RMS

Several* in vivo *models have recently been developed in* Drosophila*, zebrafish, and mice to further understand the molecular and cellular biology of RMS tumorigenesis [49–55, 91–94] (Table 5).

### 6.1. Drosophila Models in RMS

Despite the evolutionary distance, a strong conservation of genes, pathways, and regulatory molecular networks have been demonstrated between flies and humans. Many human disease genes have related sequences in* Drosophila*. In contrast to mammalian models, the generation of* Drosophila* mutants is easy, cheap, and fast.

For a better understanding of the pathogenic consequences of PAX-FKHR expression,* Drosophila* was chosen because the animal's transparent outer cuticle allows for simple real-time monitoring of muscle abnormalities elicited by PAX-FKHR, including subtle or focal changes [49]. The mutation in the myoblast fusion gene rolling pebbles (rols) was reported to dominantly suppress PAX-FKHR1-induced lethality by using a* Drosophila* model of PAX-FKHR1-mediated transformation [50]. PAX-FKHR1 signaling upregulates the TANC1 gene and blocks myoblasts from terminal differentiation. However, downregulating the TANC1 gene could cause RMS cells to lose their neoplastic state, undergo fusion, and form differentiation. This novel finding uncovered a PAX-FKHR1-TANC1 neoplasia axis collaboratively in a* Drosophila* model and loss-/gain-of-function studies in mammalian platforms [50].

### 6.2. Zebrafish Models in RMS

In contrast to other models, the short tumor onset in zebrafish allows for rapid identification of essential genes in various processes, such as tumor growth, self-renewal, and maintenance. There are other advantages for using zebrafish in the study of human disease and development including the ease and low cost of raising large numbers of fish and the highly conserved genetic and biochemical pathways between zebrafish and mammals [95].

As zebrafish RMS is highly similar to human ERMS, by using fluorescent transgenic approaches, it is more convenient to understand histogenesis and different aspects of tumorigenesis in RMS. A robust zebrafish model of RAS-induced RMS has been established, sharing two morphologic and immunophenotypic features resembling ERMS [51]. In one case, both are associated with tissue-restricted gene expression in RMS, while, in another, both comprise a RAS-induced gene signature. Cross-species microarray comparisons confirm that conserved genetic tumor-specific and tissue-restricted pathways such as RAS and p53 pathways drive RMS growth. Tg(hsp70-HRAS^G12V^) zebrafish embryos were generated to evaluate gene expression that mimics RAS pathway activation during tumorigenesis to define RAS target genes [52]. Another KRAS^G12D^-induced zebrafish embryonal RMS was utilized to assess the therapeutic effects. By the blockage of two major downstream signaling pathways, MAPK/ERK and AKT/S6K1, the model showed that inhibition of translation initiation suppresses tumor cell proliferation [52].

All the above zebrafish cancer models share similar cellular features and molecular pathways with human RMS, especially ERMS, and can demonstrate a response to therapies in a similar manner with human RMS. Thus, a tremendous opportunity is available to implement these animal models into different steps for novel targeted therapeutic development.

### 6.3. Mouse Models in RMS

Mouse models established from* in vivo* studies may contribute to the understanding of the genetic basis in tumor development and progression and may help to test the efficacies of novel antineoplastic agents.

The importance of PAX3/PAX7-FKHR fusion proteins in the progression of ARMS tumorigenesis is evident from both* in vitro* and* in vivo* studies. Transgenic mouse models generated with the PAX-FKHR fusion gene mimic the formation of human ARMS [67]. A mouse model was developed with heterozygous loss of Sufu, a tumor suppressor in Hedgehog (Hh) signaling, combined with p53 loss driven ERMS with a low penetrance (9%) [53]. These studies indicate that the models consistently forming ARMS not only need the introduction of the PAX-FKHR fusion gene but also need to be accompanied by other genetic events, such as p53 pathway disruption [54].

Compared with ARMS, ERMS models are more complex to generate because of their lower tumor penetrance and longer latency. JW41 cells from the p53/c-fos double mutant mice models resemble human ERMS cells morphologically and express similar characteristic markers [55]. The overexpression of the Wnt2 gene identified in JW41 cells confirmed in human RMS cell lines can allow for further analysis of the Wnt signaling pathway. Both of these results indicate that the downregulation of the Wnt pathway contributes to resistance to apoptosis and the inhibition of myogenic differentiation in ERMS.

Using Ptch1, p53, and/or Rb1 conditional mouse models and controlling prenatal or postnatal myogenic cell of origin, Rubin et al. demonstrated that the loss of p53 in maturing myoblasts related to tumorigenesis of ERMS [96]. They also indicated that Rb1 alteration with other oncogenic factors was strongly associated with an undifferentiated phenotype, acting as a modifier. In addition, they highlighted a subset of p53-deficient ERMSs arising from Myf6-expressing myoblasts, which had the same latency with Pax7-expressing murine satellite cells in the study.

## 7. Potential Therapeutic Targets in RMS

Detection of genomic imbalances and identification of the crucial effective genes can contribute to identifying novel potential biomarkers and relevant targets for clinical therapy in RMS.

Survivin-responsive conditionally replicating adenoviruses can regulate multiple factors (Surv.m-CRAs) [41]. Although Surv.m-CRA can efficiently replicate and potently induce cell death in RMS cells, the cytotoxic effects are more pronounced in RSC-enriched or RSC-purified cells than in RSC-exiguous or progeny-purified cells. Injections of Surv.m-CRAs into tumor nodules generated by transplanting RSC-enriched cells induce significant death in RMS cells and regression of tumor nodules. The unique therapeutic features of Surv.m-CRA include not only its therapeutic effectiveness against RMS but also its increasing effectiveness against CSC, suggesting that Surv.m-CRA may be a promising anticancer agent [41].

Rapamycin, an inhibitor of mTOR, can abrogate RMS tumor growth in a xenograft mouse model [97]. The tumors in the rapamycin-treated mice group show significant reduction of proliferation and invasiveness and induction of apoptosis. The tumor growth inhibition is simultaneous associated with the diminution of mTOR and Hh pathways, which are implicated in the pathogenesis of RMS. The results indicate that using rapamycin either alone or in combination with traditional chemotherapeutic drugs may represent a potential targeted agent for therapeutic intervention. Amplification and mutational activation of Fibroblast Growth Factor Receptor 4 (FGFR4) in RMS cells promote tumor progression [98]. Ponatinib is the most potent FGFR4 inhibitor to block wild-type RMS cell growth, mutate FGFR4 through increasing apoptosis, and suppress the FGFR4 downstream target STAT3. Ponatinib treatment slows tumor growth in RMS mouse models expressing mutated FGFR4 [98]. Ponatinib as an FGFR4 inhibitor shows potential to act as an effective therapeutic agent for RMS tumors. The expression of CXC chemokine receptor 4 (CXCR4), CXCR7, and Vascular Endothelial Growth Factor (VEGF) indicates poor prognosis in a variety of malignancies, including RMS cell lines. Most samples in a large series of clinical RMS cases show association with high expression of these antagonists regardless of their subtypes [99]. However, there are significant correlations with high expression of CXCR4 and VEGF in both ERMS and ARMS. High VEGF expression is predictive of adverse prognostic factors in RMS, as the expression of these angiogenesis factors was compared with clinicopathological parameters and prognosis. Considering their overexpression in RMS, these chemokine receptors and VEGF could provide potential molecular therapeutic targets in RMS. Some ARMS cell lines have undergone apoptosis in response to antineoplastic drugs, such as bortezomib. The proapoptotic BH3 only family member NOXA acts as an upregulated protein downstream PAX3-FKHR, resulting in increased cell sensitivity to bortezomib. Apoptosis in response to bortezomib can be reversed by shRNA knockdown of Noxa. PAX3-FKHR upregulation of Noxa creates a potential therapeutic insight into inducing apoptosis in ARMS cells. This apoptosis pathway could represent a specific targeted therapy against PAX3-FKHR-expressing ARMS cells [100].

Proton pump activation resulting from intracellular acidification may be an energetic mechanism to escape apoptosis. V-ATPase is an ATP-driven proton pump acidifying the intracellular compartment and transports protons across the plasma membrane. Esomeprazole as a V-ATPase inhibitor in the PPI administration interferes with intensive ion transporter activity of RMS cells and induces remarkable cytotoxicity. RMS expresses a higher level of V-ATPase compared with other sarcomas. Therefore, RMS should be very susceptible to esomeprazole treatment and V-ATPase could be considered as a promising selective target for treatment [101].

## 8. Conclusion and Future Prospects

For RMS, the current challenges include how to identify promising novel therapeutics and integrate them into existing therapy. For better understanding of congenital and epigenetic modifications in the development of RMS, this review summarizes the recent genome-wide studies on molecular and genetic alterations to decipher the underlying tumorigenesis mechanisms. There is a link between genetic and epigenetic alterations responsible for a variety of tumor cell growth, proliferation, differentiation, apoptosis, and therapy-resistance mechanisms. The identification of the prognostic value of the PAX-FKHR fusion status in RMS is one of the most important shifts in the lineage and the risk assessment of RMS, which exerts an oncogenic effect through multiple pathways and is incorporated into most relevant studies. The misbalanced expression of a number of miRs involved in the regulation of myogenic differentiation also implicates the tumors' escape suppressive mechanisms. Moreover, we described several* in vivo* models of RMS to explore underlying histogenesis and different aspects of tumorigenesis. Recent work on CSCs has also contributed to understanding the cell origins, refining molecular characterization, and elucidating the underlying basis of refractory responses to current adjuvant treatments of subsets of tumors. These integrative approaches can provide opportunities to identify diagnostic and prognostic biomarkers applied to the individual targeted therapy and significantly improve RMS prognosis.

## Figures and Tables

**Table 1:** Alterations of chromosome in RMS by CGH in recent 5 years.

Materials	Gain	Amplification	Loss	Deletion	Reference
25 RMS samples	2p, 12q, 6p, 9q, 10q, 1p, 2q, 6q, 8q, 15q, 18q		3p, 11p, 6p		Li et al., 2009 [16]

13 RMS cell lines	1p21.3–13.2, 1q12, 6q26–27, 7q21.3–31.1, 1q41, 2p24.3, 8q24.12, 20q13.2, 20q13.32	2p24.3 (MYCN), 8p11.23–11.21 (FGFR1), 12q13.3 (CDK4), 19q12, 20q	3p14.2–12.2, 4q27–32.3, 6p25.1–24.3, 9p24.3–24.1, 13q14.3		Missiaglia et al., 2009 [17]

57 ARMS samples		12q15, 2p24, 12q13–q14			Barr et al., 2009 [18]

128 primary RMS samples	7, 8, 11, 20	2p24.1 (MYCN), 8p11.2–p11.1 (FGFR1), 12q13.3–14.1 (CDK4), MDM2 (12q14.3–q15)			Williamson et al., 2010 [12]

26 frozen primary ERMS samples	8, 2, 11, 12, 13, 19, 20	2p21, 2q35, 2q14.2, 2q36.1, 5q35.2–q35.3, 11p11.2, 11q24.2, 12q13.3	6, 9, 10, 14, 15, 16, 18	1p36.23, 1q32.1, 3p14.2, 4q35.1–35.2, 9p21.3, 17q11.2, 22q13.31	Paulson et al., 2011 [13]

39 RMS samples	12q13.3, 12q13.3–q14.1, 12q14.1, 17q25.1	2q13.12, 12q13.3, 12q13.3–q14.1	9p12–p11.2, 10q11.21–q11.22, 14q32.33, 16p11.2, 22q11.1	1p21.1, 2q14.1, 5q13.2, 9p12, 9q12	Liu et al., 2014 [11, 15]

RMS cells derived from refractory RMS		NACA, HSD17B6, SDR9C7, RDH16, GPR182, ZBTB39, TAC3, MYO1A, NAB2, STAT6, LRP1			Park et al., 2014 [14]

20 RMS samples of Chinese patients	12q24.31, 17q25.1, 1q21.1, 7q11.23, 12q13.3–q14.1	9p13.3, 12q13.3–q14.1, 12q15, 16p13.11	5q13.2, 15q11.2, 14q32.33 (IGHG, IGHM)	1p36.33, 1p13.1, 2q11.1, 5q13.2, 8p23.1, 9p24.3, 16p11.2	Liu et al., 2014 [11, 15]

**Table 2:** The histological types of rhabdomyosarcoma.

Histological type	Predilection population	Predilection site	Risk category	Genetic change
ERMS	Infants or children under 10 years old	Head and neck region	Intermediate	LOH at chromosome 11p15.5

ARMS	Adolescents and young adults	Deep tissue of extremity	High	t(2;13)(q35;q14); t(1;13)(p36;q14)

PRMS	Adult males	Throughout the body	High	JUN (1p31), MYC (8q24), CCND1 (11q13), INT2 (11q13.3), MDM2 (12q14.3–q15)

SRMS	In the first decade of life with a second mode centered around the fifth decade	The extremities, head, and neck	Superior	SRF-NCOA2; MYOD1 mutation

ERMS: embryonal rhabdomyosarcoma.

ARMS: alveolar rhabdomyosarcoma.

PRMS: pleomorphic rhabdomyosarcoma.

SRMS: spindle cell/sclerosing rhabdomyosarcoma.

**Table 3:** Studies identifying stem cells in RMS.

Marker/substrate	Source	Stem cell gene	Functional characterization	Reference
CD133	Orthotopic xenograft model	OCT4, NANOG, c-MYC, PAX3, and SOX2	Correlating with poor overall survival	Walter et al., 2011 [38]

PAX-FKHR	Bone marrow of C57BL/6 mice	Myf5, MEF2, MyoD, and myogenin	Determining the molecular, myogenic, and histologic phenotype of ARMS	Ren et al., 2008 [39]

V-ATPase	RD cell line	NANOG and OCT3/4	Driving mechanisms of a reduced sensitivity to anticancer drugs and activities related to invasion and metastasis	Salerno et al., 2014 [40]

Surv.m-CRAs	KYM-1 cell line (FGFR3-positive)	FGFR3	Therapeutic effectiveness against all cell populations and increased effectiveness against CSCs	Tanoue et al., 2014 [41]

**Table 4:** MicroRNAs involved in myogenesis and RMS development.

miRNA	Expression	Target	Function	Reference
miR-1 and miR-133a	Downregulation	MYH9	Myogenic miRNA, inhibit differentiation and promote proliferation in myogenesis, cytostatic	Rao et al., 2010 [42]

miR-206	Downregulation	cMET	Promote differentiation and proliferation in myogenesis	Yan et al., 2009 [43]

miR-29	Downregulation	HADC4, YY1, EZH2	Promote stabilization of RMS phenotype	Marchesi et al., 2014 [44]

miR-450-5p	Downregulation	ENOX, PAX9	Promote differentiation and progression	Sun et al., 2014 [45]

miR-203	Downregulation	JAK, STAT, Notch	Inhibit differentiation and proliferation in myogenesis, tumor suppressor	Diao et al., 2014 [46]

miR-214	Downregulation	N-Ras	Inhibit tumor cell growth and induce myogenic differentiation and apoptosis	Huang et al., 2014 [47]

miR-183	Upregulation	EGR1	Promote migration and metastasis	Sarver et al., 2010 [48]

**Table 5:** *In vivo* animal models of RMS.

Model	Target		Model origin	Reference
	Inactivation	Expression		
*Drosophila*		PAX-FKHR	Bipartite Gal4-UAS expression system	Galindo et al., 2006 [49]
	PAX7-FOXO1	rols	Chromosomal deletion, Df(3L)vin5	Avirneni-Vadlamudi et al., 2012 [50]

Zebrafish	rag2 promoter	c-Myc	kRAS^G12D^	Chen and Langenau, 2011 [51]
	MAPK/ERK and AKT/S6K1	PD98059, TPCK	Tg(hsp70-HRAS^G12V^)	Le et al., 2012 [52]

Mouse	Sufu	N-myc, Sfrp1, Ptch2, and cyclin D1	Sufu^+/−^	Lee et al., 2007 [53]
	Ink4a/Arf and Trp53	Pax3:FKHR	Pax3^P3Fp/wt^	Keller et al., 2004 [54]
	Wnt/*β*-catenin signaling pathway	Wnt2	p53^−/−^/c-fos^−/−^	Singh et al., 2010 [55]

## Abbreviations

RMS: Rhabdomyosarcoma
ARMS: Alveolar rhabdomyosarcoma
ERMS: Embryonal rhabdomyosarcoma
PRMS: Pleomorphic rhabdomyosarcoma
SRMS: Sclerosing/spindle cell rhabdomyosarcoma
miRNA, miR: MicroRNA
CSC: Cancer stem cell
MSC: Mesenchymal stem cell.
